# Phenotypic Diversity of a Leafroller *Archips podana* (Lepidoptera, Tortricidae) Does Not Change along an Industrial Pollution Gradient

**DOI:** 10.3390/insects14120927

**Published:** 2023-12-05

**Authors:** Mikhail V. Kozlov

**Affiliations:** Department of Biology, University of Turku, 20014 Turku, Finland; mikoz@utu.fi

**Keywords:** male genitalia polymorphism, steel smelter, phenotypic diversity, pheromone trapping, population abundance, sulphur dioxide

## Abstract

**Simple Summary:**

Populations of the large fruit-tree tortrix *Archips podana* consist of a mixture of four morphs that differ in male genitalia traits, ecology and sexual behaviour. Here, I tested whether these morphs differently respond to pollution. I found that the within-population diversity of *A. podana* around the industrial city of Lipetsk in central Russia correlated neither with the distance to the nearest industrial polluter nor with the number of moths captured by pheromone traps. Thus, the four phenotypes of *A. podana* similarly respond to pollution.

**Abstract:**

Morphological polymorphism offers rich opportunities for studying the eco-evolutionary mechanisms that drive the adaptations of local populations to heterogeneous and changing environments. In this study, I explore the association between pollution load, abundance of large fruit-tree tortrix *Archips podana* and its within-species diversity (expressed in the presence of apical and/or lateral prongs on the phallus in male genitalia) across 26 study sites located 0.5 to 31 km from the industrial city of Lipetsk in central Russia. The Shannon diversity index, calculated from the frequencies of four morphs, correlated neither with the distance to the nearest industrial polluter (a proxy of pollution load) nor with the number of moths captured by pheromone traps (a measure of population abundance). The statistical power of the correlation analysis was sufficient (67%) to detect a medium effect (i.e., Pearson correlation coefficient with an absolute value of 0.40), if it existed. I conclude that the four phenotypes of *A. podana* do not differ in tolerance to industrial pollution and similarly respond to pollution-induced environmental disturbance. This is the first study of industrial pollution impacts on within-species diversity of insects expressed in the discrete traits of their male genitalia.

## 1. Introduction

Polymorphism, i.e., the co-existence of two or more clearly different morphs (alternative phenotypes) in the population of a species, offers rich opportunities for studying the eco-evolutionary mechanisms driving the adaptations of local populations to heterogeneous and changing environments [1,2,3]. These opportunities are especially important in the face of rapid environmental change, which is increasingly recognized as a crucial evolutionary force [4,5].

The analysis of temporal changes in the diversity and abundance of different groups of biota near industrial polluters suggests that evolutionary adaptation to the impact of pollution is a general phenomenon [6]. Nevertheless, the rate of this adaptation is currently insufficient to counterbalance the adverse effects of human-induced disturbance on biodiversity, and the decrease in diversity is commonly seen as a general ecosystem response to stress [7,8].

The published evidence on the decline in biodiversity with an increase in industrial pollution is dominated by species-level studies [6,9,10]. These studies demonstrated that species differ in their ability to sustain a polluted environment, and that this ability can be predicted from species’ life history traits [11]. Thus, some character states present in the ancestral (pristine) environment (termed exaptations [12]) facilitate colonisation of and survival in a novel (polluted) environment.

Previous studies addressing exaptations of certain phenotypes (morphs) of insects to polluted environments have focused on industrial melanism, i.e., on an increased occurrence of melanic individuals in heavily polluted regions [13]. Melanic morphs, which were described in several dozens of insect species well before the industrial revolution, appeared better adapted to coal-polluted urban environments relative to typical (i.e., non-melanic) morphs [14]. The best studied insects showing these exaptations are the peppered moth *Biston betularia* [15,16] and the two-spot ladybird *Adalia bipunctata* [17].

Surprisingly, increased frequencies of melanic morphs have never, to my knowledge, been observed in insect populations living close to large industrial polluters emitting huge amounts of sulphur dioxide, fluorine and trace elements in ambient air, although these emissions greatly affect the performance, abundance and diversity of many insects [6,18,19]. Three studies conducted in the impact zone of a copper–nickel smelter in Monchegorsk, NW Russia, did not discover pollution-related changes in the occurrence of melanic morphs of noctuid moths [20], leaf beetle *Chrysomela lapponica* [21] and brassy tortrix *Eulia ministrana* [22]. I am not aware of any study of the industrial pollution impacts on within-species diversity in insects expressed in discrete traits of their genitalia.

At the same time, rapid genetic and/or biochemical adaptations to pollution were reported in many insect species [23,24,25], suggesting that insect assemblages do respond dynamically to major anthropogenic evolutionary challenges. This finding stresses the urgent need to overcome a gap between studies exploring changes in the genetic structure of insect populations exposed to environmental pollution and those exploring pollution effects on insect morphological or functional traits. It seems apparent that meeting this goal would be greatly facilitated by the discovery of a species whose alternative phenotypes (morphs) differ in their tolerance to pollution.

The study described below was designed to check whether the large fruit-tree tortrix *Archips podana* (Scopoli, 1763) could serve as a model polymorphic species for investigation of pollution-driven microevolution in insect populations. In this study, I explore the association between the distance from the nearest industrial polluter around the city of Lipetsk in central Russia, abundance of *A. podana* and its phenotypic diversity. The four morphs of *A. podana* differ in sexual behaviour [26] and host plant preference [27]. Industrial pollution was reported to disrupt pheromone communication in insects [28], thus affecting sexual selection [29], and change both the composition of plant communities [9,10] and host plant quality for herbivores [18,30]. Therefore, there was a good reason to expect that these morphs differently respond to industrial pollution, and therefore the intraspecific diversity of *A. podana*—in parallel with the species-level diversity of moths and butterflies [11,20,31]—declines with an increase in pollution load.

## 2. Materials and Methods

### 2.1. Study Species

*Archips podana* is a relatively large (wing span 18–26 mm) leafroller widely distributed in Europe. Its larvae feed on a variety of deciduous trees and shrubs, including apple and pear [32]. In the study region (central Russia), moths are on wing from early June to early July and then from early August to mid-September. Females lay eggs in groups of 50–100 on the leaf surface. Larvae spin a fine web and feed on the underside of a leaf. Second and third instars feed on the surface of a fruit, often webbing leaves to it. Larvae of the second generation overwinter in the second instar and continue development in the following spring. Pupation occurs in the larval shelter [33,34,35].

**Figure 1:**
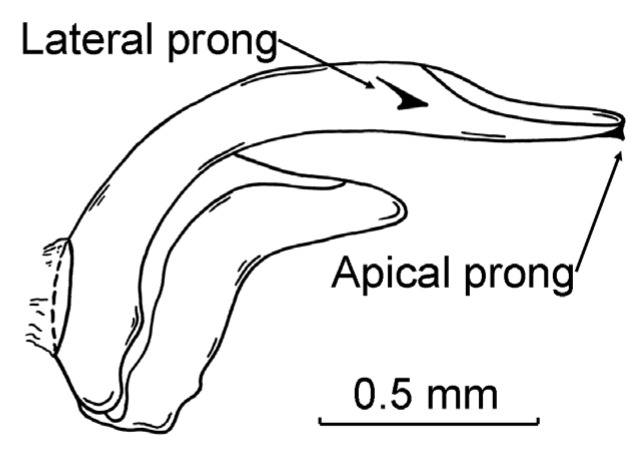
Traits of phallus in male genitalia of *Archips podana* used to distinguish the four morphs.

*Archips podana* is polymorphic in male genitalia traits which have long been considered as diagnostic for *Archips* species [36]. This polymorphism was discovered in the 1980s [37] and has been intensively studied since then as a morphological marker of many ecological and physiological traits of *A. podana* males [26,38,39,40]. The four morphs differ by the presence or absence of a ventrally directed prong at the apex of the phallus and a laterally directed prong on the left side of the phallus, ca. 0.7 of its length counting from the base (Figure 1). These two characters are heritable [41], and males of all phenotypes may occur in a progeny of the same parents (M. Kozlov, pers. obs.).

### 2.2. Study Area

Lipetsk (52°37′ N, 39°36′ E) is a medium-sized industrial city located 450 km SE of Moscow. In 1991, 92% of aerial emissions in Lipetsk emerged from the metallurgical industry [42]. The main polluters were a steel factory, a metallurgical factory combined with a tractor plant, and an agglomeration factory, which were established in 1902, 1935, and 1944, respectively [43]. The primary pollutants emitted by the first two factories were sulphur and nitrogen oxides, whereas the third factory emitted large amounts of dust. No other strong emission sources existed within 50 km from Lipetsk during the study period.

In 1991, all industries located in Lipetsk jointly emitted 32,500 metric tons (t) of sulphur dioxide, 23,300 t of nitrogen oxides and 41,000 t of dust in ambient air [42]. Emission of trace elements had not been reported; nevertheless, the fine roots of the Scots pine (*Pinus sylvestris* L.) near the steel factory contained 10 times more zinc and 25 times more cadmium compared with a forest located 17 km away [44]. Despite the decline in emissions during the past decades, the peak concentration of sulphur dioxide in the ambient air of Lipetsk in 2020 reached 1100 μg m^−3^ [45].

### 2.3. Sampling

Males of *A. podana* were collected using cardboard delta traps with a sticky insert from 26 sites (Table S1) located 0.5 to 31 km from the nearest of the three polluters specified above. This design assured the coverage of the entire impact zone of these polluters, because sites located 17–34 km from Lipetsk represent regional pollution background [46,47]. The traps (five per site) were exposed within forest shelterbelts from 5 June to 12 July 1992. The bait (a piece of rubber pipe) contained 1.0 mg of (E)-11-tetradecenyl acetate and 0.5 mg of (Z)-11-tetradecenyl acetate (trademark AO—81, Flora Inc., Tartu, Estonia). In total, 12 of the 130 deployed traps were vandalised; thus, moth abundance was estimated from 118 traps (Data S1). Furthermore, the abdomina of all *A. podana* males in 11 traps were destroyed by predatory and saprophagous animals; thus, polymorphism was measured in samples from 107 traps only. For this purpose, intact abdomina of *A. podana* were cleaned of glue in medicinal ether, macerated in a 10% solution of KOH and classified to one of four morphs (Figure 1) based on phallus morphology (no prongs; only apical prong(s) present; only lateral prong(s) present; both prongs present) using a stereomicroscope (Data S1).

### 2.4. Statistical Analysis

For each sample, I calculated the proportion of each morph and the Shannon diversity index based on the natural logarithms of these proportions. Ten samples consisting of 1–5 individuals were excluded from the analyses, because these samples yielded outlying values of morph frequencies and diversity. The numbers of moths captured by pheromone traps and the Shannon diversity index were compared among sites by ANOVA. Following a widely used practice [10,48,49], the log_10_-transformed distance from the nearest polluter was used as a proxy of pollution load. The relationships between site-specific values of all variables (pollution load, number of captured moths, frequencies of individual morphs, and Shannon diversity index) were quantified by calculating Pearson product–moment correlation coefficients. The direction from the polluter was not included in the analysis, because pollutant deposition around Lipetsk similarly decreased in the four compass directions [47].

All correlations reported in this study (see below) appeared non-significant. Therefore, I estimated the statistical power of my analysis, i.e., the likelihood of a significance test detecting a correlation in a sample when it occurs in the study population [50]. For calculation of statistical power (power.phs.wakehealth.edu/index.cfm?calc=cor, accessed on: 10 November 2023: α = 0.05, one-tailed test), I used |*r*| = 0.40 obtained by averaging 1446 absolute values of correlation coefficients between the measures of pollution load and diverse biotic variables [10].

## 3. Results

**Figure 2:**
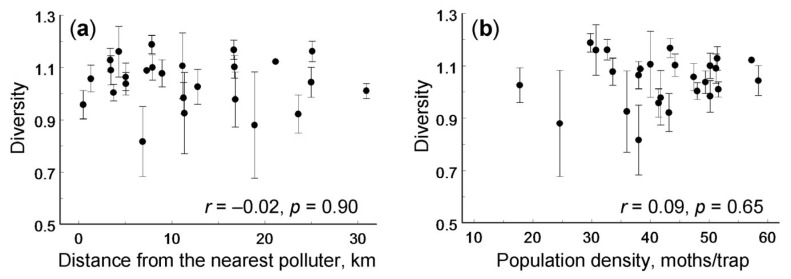
Diversity of *Archips podana* (mean ± SE, median sample size 4 traps per site) in relation to (**a**) distance to the nearest industrial polluter and (**b**) population abundance.

The abundance of *A. podana* varied among study sites (*F*_25,92_ = 2.58, *p* = 0.0006); however, this variation was not explained by the log_10_-transformed distance to the nearest polluter (*r* = −0.03, *n* = 26 sites, *p* = 0.89). The frequencies of individual morphs correlated neither with the distance to the nearest polluter nor with the number of males captured by pheromone trap (Table 1). The Shannon diversity index did not vary among study sites (*F*_25,74_ = 0.73, *p* = 0.81) and did not correlate with either of these explanatory variables (Figure 2). The statistical power of the correlation analysis, i.e., the probability of detecting a medium-size effect (|*r*| = 0.40), was 67%.

## 4. Discussion

### 4.1. How Robust Are the Results?

A chance to detect a medium (as classified by [51]) effect in my data, if it exists in nature, was 67%. This statistical power is reasonably high relative to an average of 23–47% calculated for studies reported in ecology, evolution and behaviour journals two to three decades ago [52,53], and especially relative to 15% across the studies summarised by current meta-analyses in ecology and evolutionary biology [54]. The statistical power of 67% is exceptionally high for correlation studies conducted in industrial pollution gradients, because only 25% of them had a chance to detect a medium effect [10]. Thus, my conclusions on the absence of association between the pollution load, the abundance of *A. podana* and the level of its polymorphism are robust if a medium or large effect is expected. By contrast, the chances of detecting a small (|*r*| = 0.10) effect were only 12%.

### 4.2. Potential Drivers of the Phenotypic Structure of Archips podana Populations

Previous studies of pollution impacts on insect polymorphism mainly employed colour morphs and attributed the observed changes in morph frequencies to their thermal preferences; selection by visually hunting predators, primarily birds; sexual attraction; and immunity [13,14,15]. By contrast, no direct adaptive explanation exists for two morphological characters in male genitalia (i.e., apical and lateral prongs on the phallus), the occurrence of which defines the phenotypes of *A. podana*. This naturally hampers the interpretation of my results.

In 1991, the abundance of *A. podana* near the city of Lipetsk was more than two-fold greater relative to distant (unpolluted) sites, whereas moth size declined while approaching the polluters [34]. This decrease in moth size could be attributed to the toxic effects of pollutants, whereas an increase in moth abundance implies a decline in an overall mortality risk due to predation or parasitism (the enemy-free space hypothesis [18,30]). Thus, despite pollution impacts near the city of Lipetsk affected the size and abundance of *A. podana*, the phenotypic structure of this species did not change. The latter result suggests a similar tolerance of *A. podana* morphs to industrial pollutants and the absence of differential selection of morphs by natural enemies.

Pollution gradients are often accompanied by climatic gradients arising due to urban heat island effect [55] and alterations of microclimate caused by adverse pollution effects on vegetation [10,56]. Across Europe, the proportion of *A. podana* males with apical prong(s) on the phallus decreases, while the proportion of males with lateral prong(s) on the phallus increases from north-west to south-east [38]. The clinal changes in morph frequencies in ectothermic animals are often associated with climatic gradients [57,58]. However, the absence of differences in the phenotypic structure of *A. podana* populations between proximate (urban and suburban) and distant (rural) sites suggests that morphs of *A. podana* have similar climatic requirements; therefore, the geographic variation in morph frequencies in *A. podana* populations is likely driven by factors other than temperature.

To conclude, the phenotypic structure of *A. podana* populations does not change along an industrial pollution gradient. Consequently, this species could not serve as a model for studies of phenetic changes in polymorphic insect populations caused by pollution.

### 4.3. Should Old Data Be Published?

The data reported in this study were collected more than 30 years ago in the course of an environmental project addressing the impacts of industrial pollution on insects. The results were not published at that time because of the negative attitude of the scientific community towards non-significant results which was prevalent in the 1990s. The amount of sulphur dioxide emitted by metallurgical industries in Lipetsk has declined by one-third since then [59], and so from an environmental perspective, these old data are of limited interest. However, these data can still be used for testing eco-evolutionary hypotheses addressing pollution impact on phenotypic diversity of insect populations.

The delayed publication of data showing no statistically significant effect, which is widespread in ecological and evolutionary studies, results in a decline in the cumulative effect size over time [60,61]. The acute need for obtaining unbiased estimates of effect sizes [54] further justifies the publication of old data. I hope that this publication will stimulate a debate on relationships between animal polymorphism and industrial pollution.

## Figures and Tables

**Table 1:** Pearson correlation coefficients between frequencies of *Archips podana* morphs, log_10_-transformed distance to the nearest polluter and population abundance (n = 26 sites).

Morph Characteristics	Distance to Polluter		Population Abundance	
	*r*	*p*	*r*	*p*
No prongs	0.28	0.17	−0.18	0.38
Apical prong(s) only	0.08	0.71	−0.31	0.12
Lateral prong(s) only	−0.03	0.88	0.26	0.20
Both prongs	−0.13	0.52	0.07	0.75

## Data Availability

All data are included in Supplementary Materials.

## Supplementary Materials

The following supporting information can be downloaded at: https://www.mdpi.com/article/10.3390/insects14120927/s1, Table S1: Coordinates and elevations of industrial polluters and study sites, and distances to the nearest polluter; Data S1: Numbers of *A. podana* males captured by pheromone traps (total and by morphs).
