# Supplementary material for: Phenotypic Diversity of a Leafroller Archips podana (Lepidoptera, Tortricidae) Does Not Change along an Industrial Pollution Gradient
Source: Insects. 2023 Dec 5;14(12):927. doi: 10.3390/insects14120927 (PMC10743578; doi:10.3390/insects14120927)
Supplement: Supplementary file 1 [file insects-14-00927-s001.zip › insects-2747813-supplementary.pdf]

Phenotypic diversity of a leafroller *Archips podana* (Lepidoptera, Tortricidae) does not change along an industrial pollution gradient

Mikhail V. Kozlov [1,*]

[1] Department of Biology, University of Turku, Turku, Finland
* Correspondence: mikoz@utu.fi

Supplementary material

Table S1. Coordinates and elevations of industrial polluters and study sites, and distances to the nearest polluter

Locality [1]	Latitude, N	Longitude, E	Elevation, m	Distance to polluter, km
Polluter A	52° 37' 48"	39° 39' 42"	110	-
Polluter B	52° 33' 23"	39° 35' 50"	120	-
Polluter C	52° 33' 46"	39° 41' 59"	120	-
Site 1	52° 34' 10"	39° 08' 31"	200	30.90
Site 2	52° 34' 51"	39° 13' 50"	220	24.98
Site 3	52° 35' 45"	39° 17' 28"	220	21.15
Site 4	52° 36' 10"	39° 21' 44"	210	16.73
Site 5	52° 36' 18"	39° 26' 57"	190	11.35
Site 6	52° 35' 48"	39° 30' 00"	154	7.93
Site 7	52° 36' 19"	39° 35' 57"	110	5.05
Site 8	52° 34' 25"	39° 32' 26"	170	4.30
Site 9	52° 32' 02"	39° 33' 50"	120	3.38
Site 10	52° 30' 39"	39° 35' 49"	130	5.07
Site 11	52° 29' 12"	39° 39' 43"	120	8.95
Site 12	52° 26' 57"	39° 41' 49"	130	12.79
Site 13	52° 31' 58"	39° 29' 20"	170	7.84
Site 14	52° 30' 12"	39° 27' 24"	170	11.25
Site 15	52° 27' 56"	39° 24' 10"	150	16.67
Site 16	52° 28' 14"	39° 21' 27"	170	18.89
Site 17	52° 26' 28"	39° 16' 48"	180	25.09
Site 18	52° 33' 42"	39° 43' 08"	120	1.29
Site 19	52° 32' 57"	39° 44' 45"	120	3.47
Site 20	52° 31' 47"	39° 47' 32"	120	7.32
Site 21	52° 30' 24"	39° 50' 06"	120	11.15
Site 22	52° 38' 02"	39° 39' 26"	120	0.49
Site 23	52° 39' 23"	39° 41' 43"	140	3.75
Site 24	52° 41' 28"	39° 40' 35"	130	6.88
Site 25	52° 46' 35"	39° 43' 18"	140	16.81
Site 26	52° 49' 49"	39° 45' 29"	120	23.60

[1] Polluter A: steel factory; Polluter B: metallurgical factory combined with a tractor plant; Polluter C: agglomeration factory.

Data S1 Numbers of *Archips podana* males captured by pheromone traps (total and by morphs).

Column 1: site (for position, consult Table S1).
Column 2: trap (numbers nested within a site).
Column 3: total number of *A. podana* males captured by the trap.
Column 4: number of males without prongs on phallus.
Column 5: number of males with apical prong(s) only.
Column 6: number of males with lateral prong(s) only.
Column 7: number of males with both types of prongs on phallus.

Full stops indicate missing data. Columns 4-7 do not include the individuals, the genitalia of which were damaged by predatory or saprophagous animals.

1 1 63 1 16 5 4
1 2 43 0 14 5 3
1 3 47 0 8 7 3
1 4 42 0 10 5 6
1 5 63 0 20 11 10
2 1 71 . . . .
2 2 54 3 13 3 3
2 3 50 0 18 5 5
2 4 65 4 16 3 5
2 5 52 0 12 12 4
3 1 59 . . . .
3 2 . . . . .
3 3 47 1 11 3 8
3 4 71 . . . .
3 5 52 . . . .
4 1 60 1 19 5 9
4 2 13 0 0 0 1
4 3 57 2 11 4 5
4 4 55 1 15 6 7
4 5 36 1 14 3 4
5 1 42 1 10 4 3
5 2 60 1 10 8 9
5 3 36 0 11 0 3
5 4 34 1 5 0 9
5 5 8 . . . .
6 1 57 1 3 1 2
6 2 45 0 3 4 3
6 3 29 0 13 4 6
6 4 52 1 15 4 8
6 5 68 0 9 5 8
7 1 37 0 9 6 6
7 2 43 0 12 7 10
7 3 29 0 8 2 3
7 4 . . . . .
7 5 43 1 7 3 8
8 1 36 1 2 1 2
8 2 35 0 5 2 2
8 3 43 2 9 2 3
8 4 9 0 3 0 2
8 5 . . . . .
9 1 53 0 11 5 6
9 2 47 0 4 3 3
9 3 47 1 19 12 9
9 4 75 3 16 9 15
9 5 35 . . . .
10 1 58 0 14 9 5
10 2 44 1 12 2 14
10 3 38 1 7 8 4
10 4 53 0 18 9 5
10 5 54 0 19 7 7
11 1 40 0 11 7 3
11 2 33 0 9 3 3
11 3 35 0 7 4 4
11 4 31 1 6 4 2
11 5 29 1 7 4 9
12 1 6 0 3 0 0
12 2 1 0 0 0 0
12 3 4 0 0 0 2
12 4 29 0 7 2 3
12 5 49 0 13 10 12
13 1 29 2 9 4 3
13 2 33 0 3 1 0
13 3 17 0 3 0 1
13 4 36 2 11 4 7
13 5 34 2 11 4 2
14 1 53 1 15 2 5
14 2 51 1 14 8 7
14 3 50 0 12 4 4
14 4 51 0 12 6 7
14 5 46 0 15 3 3
15 1 19 4 6 2 2
15 2 47 1 11 4 10
15 3 43 1 16 8 6
15 4 57 1 21 12 10
15 5 51 0 0 0 0
16 1 2 0 1 1 0
16 2 15 0 0 9 2
16 3 60 1 27 10 11
16 4 5 0 0 0 0
16 5 41 0 12 12 11
17 1 35 2 7 2 3
17 2 28 0 4 4 4
17 3 . . . . .
17 4 35 2 10 2 9
17 5 . . . . .
18 1 58 0 17 4 12
18 2 42 2 10 5 9
18 3 43 0 16 5 8
18 4 50 0 10 6 10
18 5 44 0 12 4 9
19 1 54 1 17 6 13
19 2 46 2 13 10 4
19 3 49 1 10 9 5
19 4 54 0 16 6 3
19 5 53 0 15 10 10
20 1 . . . . .
20 2 29 0 7 5 6
20 3 24 0 2 2 0
20 4 . . . . .
20 5 62 0 10 14 14
21 1 39 0 8 1 4
21 2 28 0 3 0 0
21 3 . . . . .
21 4 38 1 12 8 11
21 5 55 2 10 8 9
22 1 54 0 20 7 9
22 2 55 0 20 2 8
22 3 39 0 11 3 11
22 4 53 0 7 4 8
22 5 6 0 1 1 0
23 1 48 1 18 3 10
23 2 45 . . . .
23 3 51 0 15 10 7
23 4 49 0 0 0 0
23 5 47 0 8 2 10
24 1 . . . . .
24 2 . . . . .
24 3 . . . . .
24 4 27 0 4 3 0
24 5 49 1 11 2 2
25 1 48 0 18 3 3
25 2 53 0 8 2 5
25 3 36 2 9 4 4
25 4 30 0 7 3 2
25 5 . . . . .
26 1 52 0 26 2 5
26 2 41 0 6 3 11
26 3 51 0 18 5 7
26 4 27 0 11 4 4
26 5 45 0 8 5 5
